# Ireland's takeover of private hospitals during the COVID-19 pandemic

**DOI:** 10.1017/S1744133121000189

**Published:** 2021-05-18

**Authors:** Julien Mercille, Brian Turner, Donnacha Seán Lucey

**Affiliations:** 1 School of Geography, University College Dublin, Dublin, Ireland; 2 Department of Economics, University College Cork, Cork, Ireland; 3 College of Business and Law, University College Cork, Cork, Ireland

**Keywords:** COVID-19, health policy, Ireland

## Abstract

In Ireland, the coronavirus disease 2019 (COVID-19) pandemic has led to a total of 230,599 cases of infection as on 20 March 2021, and 4323 deaths. Although the Irish hospital network has not been overwhelmed, it has faced pressures, with a total of 13,313 persons hospitalised, including 1402 admitted to the intensive care unit. Out of caution, in spring 2020, in anticipation of possible surges in hospitals in light of international experience, the Irish government reached an agreement with private hospitals to access their capacity for three months to alleviate pressure on the public system, as part of its comprehensive response to the pandemic. This piece analyses the agreement with private hospitals, based on the legally binding Heads of Terms of the agreement, which were signed by the parties, along with publicly reported details from media reports and Oireachtas (parliamentary) committee hearings. We argue that although the new relationship could, in theory, have paved the way to the nationalisation of the whole hospital system, in fact, the experiment is best interpreted as a lost opportunity to integrate and simplify Ireland's hospital system.

In Ireland, the coronavirus disease 2019 (COVID-19) pandemic has led to a total of 230,599 cases of infection as on 20 March 2021, or 4842.6 per 100,000 population, as well as 4323 deaths (Health Protection Surveillance Centre, 2021). Although the Irish hospital network has not been overwhelmed as in Spain, Italy and New York City, it has nevertheless faced pressures, with a total of 13,313 persons having been hospitalised, including 1402 admitted to intensive care unit (ICU) (Health Protection Surveillance Centre, 2021). Out of caution, in spring 2020, in anticipation of possible surges in hospitals in light of international experience, the Irish government reached an agreement with private hospitals to access their capacity for three months to alleviate pressure on the public system, as part of its comprehensive response to the pandemic (Burke *et al*., 2020). This piece analyses the agreement with private hospitals, based on the legally binding Heads of Terms of the agreement, which were signed by the parties (Government of Ireland, 2020), along with publicly reported details from media reports and Oireachtas (parliamentary) committee hearings. We argue that although the new relationship could, in theory, have paved the way to the nationalisation of the whole hospital system, in fact, the experiment is best interpreted as a lost opportunity to integrate and simplify Ireland's hospital system.

The Irish health system contains complex interactions between public and private funding and delivery (Burke *et al*., 2018*b*; European Commission, 2019; Independent Review Group, 2019; Mercille, 2018*a*, 2019; Mercille and O'Neill, 2020; Turner and Smith, 2020). Nowhere is this more obvious than in the hospital sector. Acute hospitals are divided into two main categories according to their main source of funding. There are some 50 public hospitals (some run directly by the Health Service Executive – HSE, others by non-profit organisations) whose funding largely comes from public sources, and there are 19 private hospitals whose funding is largely obtained from private sources (mostly from private health insurance) (Mercille, 2018*b*). Private hospitals, however, do not have emergency room facilities comparable to public hospitals and tend to specialise in elective procedures (e.g., orthopaedics).

To complicate matters, private patients are often treated in public hospitals, subsidised by the State through tax relief on private health insurance premiums (Turner, 2015), while public patients who have had long waiting times are sometimes treated, at the expense of the State, in private hospitals under the National Treatment Purchase Fund.

Prior to the onset of the pandemic, the Irish hospital system had lower capacity than many other European and industrialised countries. In 2017, Ireland had 3.0 hospital beds per 1000 population, compared with an OECD (Organisation for Economic Co-operation and Development) average of 4.7, and the highest bed occupancy rate in the OECD, at 94.9%, compared with an OECD average of 75.2% and higher than the recommended safe level (OECD, 2019).

This capacity shortfall has been acknowledged by an all-party parliamentary committee on the future of healthcare (Burke *et al*., 2018*a*; Committee on the Future of Healthcare, 2017) and by a capacity review of the health sector (Department of Health, 2018). These forecast that at least 2590 additional inpatient hospital beds are needed, along with significant extra human resources across the health service over the lifetime of the 10-year plan for the Irish health system (known as Sláintecare).

As a result of concerns over the capacity of the public hospital system to cope with the pandemic, in April 2020, the HSE, which provides public health services nationally, entered into a temporary agreement with the private hospitals, which enabled the HSE to access the capacity of the private hospital system for an initial period of three months, with an option to extend this if needed (which was not taken up). The agreement cost the government over €300 million (Special Committee on Covid-19 Response, 2020*a*, p. 43), paid to private hospital owners.

The increased role of the State in private hospitals provided a glimpse of how the pandemic could have re-shaped Irish healthcare along the lines of a national health service as in the United Kingdom. Indeed, while Ireland and the United Kingdom share a deep historical relationship, their health systems have followed very different paths after the Second World War. The war in Britain brought the establishment of the Emergency Health Services which saw the State take over beds in privately run voluntary hospitals along with the integration of hospital services across the poor law, municipal and charitable sectors. Ireland's modern-day private hospitals are not directly analogous to Britain's 1940s’ voluntary hospitals; the former are stand-alone private entities with shareholders while the latter were charitable bodies based on philanthropic effort and honorary medical staffs who contributed their services *gratis*. Yet pre-1940s’ British health services were marked by complexity and disaggregation, where access and entitlement greatly varied by region and sector, which made attempts at reform desultory and ineffective. The path to the NHS was by no means an inevitable or logical progression (Webster, 2002). The extensive state intervention brought about by the experience of war had a long-lasting impact on the United Kingdom's health services which may not have otherwise transpired. For the first time, much of the population was entitled to free healthcare which helped to set a precedent for the universal, free at the point of contact service which arrived in 1948 with the NHS's establishment. Yet Ireland's wartime experience was largely benign and, unlike in Britain, did not act as a catalyst for healthcare reform. Insuperable vested interest groups including the medical profession, voluntary hospitals and religious authorities resisted nationalisation or universalism. This was especially evident when an alliance of the Catholic Church and medical profession prevented the introduction of the 1951 Mother and Child scheme which proposed to give free primary care to mothers and their children. Ireland's failure to develop a universal healthcare system was embedded in the country's rurality, weakness of a social democratic movement and prominence of the Catholic Church in policy making. In turn, the lack of universal care and mixed provision of state, voluntary and private providers makes Ireland an outlier in European healthcare states (Lucey, 2015; Wren and Connolly, 2019).

The agreement with private hospitals in Ireland during the COVID-19 pandemic did enable patients to be transferred from public hospitals to private hospitals, or be admitted directly as public patients, which reduced pressure on the public health system, while no private work was allowed to be admitted to the private hospitals after the date of commencement of the agreement (30 March 2020), although continuity of care for inpatients already in those hospitals at that date, or existing patients who required treatment, was provided for. However, the government takeover of the entire private hospital network encountered three key problems that ultimately led the government not to renew it:^1^ (1) its price tag was very high; (2) the private hospitals remained significantly under-utilised for the duration of the agreement; (3) only 291 of the approximately 550 private-only consultants took up the type A contracts proposed by the agreement,^2^ citing concerns about continuity of care for private patients and access to their outpatient rooms (Special Committee on Covid-19 Response, 2020*b*, p. 71).

Fortunately, those problems did not result in significant negative consequences for care because COVID-19-related hospitalisations remained manageable. However, this emboldened critics of the agreement, from the political left and right, who emphasised that the government had paid too much for a takeover whose utilisation was never maximised.

Private hospital consultants were especially vocal in their criticisms of the deal (Special Committee on Covid-19 Response, 2020*a*). They were dissatisfied that the agreement only offered them ‘Type-A’ contracts^3^ that provided for public work only for the duration of the agreement. Many stories appeared in the national media claiming that this jeopardised their existing private patients. However, in fact, the agreement contained steps to ensure continuity of care, including pro bono work by private consultants who had not signed the deal (Breslin, 2020, pp. 35, 40–41; Woods, 2020, pp. 36–37). Moreover, the government had decided to suspend non-essential elective procedures, which would inevitably lower private hospitals’ activity, from late March to early May. But this decision was due to COVID-19 and was not an effect of the hospital agreement.

Nevertheless, one central problem with the agreement is that its negotiation had largely excluded the consultants themselves. It had been struck between the government and hospital owners, and was more or less presented to consultants as a fait accompli, which did nothing to assuage their dissatisfaction. The consultant representatives subsequently claimed that the lack of engagement was the agreement's ‘big failure’ and any future measures would need greater ‘tripartite engagement’ between the health service management, private hospital associations and private practice consultants (Varley, 2020). Powerful vested interest groups, such as private consultants, have been identified in the wider literature as often blocking progress toward reform, not just in Ireland but health systems generally (Connolly and Wren, 2019, pp. 96–97) and potential consultant implacability risked slowing the initial agreement, which was an emergency response. However, the failure of many consultants to participate indicates that some degree of engagement with this key stakeholder group is necessary.

As the agreement was about to run its course, the government cabinet was unwilling to renew it on the same terms, citing financial reasons, and let it expire (Wall, 2020*b*). However, it decided to negotiate a ‘Surge II agreement’, drawing lessons from the original deal's problems. Government officials are of the opinion that, first, the new agreement will need to be more targeted (Wall, 2020*a*). For example, it should identify specific types of procedures that private hospitals could accomplish efficiently instead of taking over the private hospital system as a whole; bilateral deals with certain private hospitals could be effective in this regard. These payments will likely be made through the National Treatment Purchase Fund and will reduce costs by leveraging private capacity in line with changing requirements. Consultants should also be included in future negotiations to increase participation and prevent legal action on their part (Cahill, 2020).

Those developments are happening within a context of increasing hospital waiting lists, a longstanding problem in Ireland now worsened by COVID-19 due to the reduction in the number of regular procedures carried out during the pandemic. Figures from the National Treatment Purchase Fund show that, as of June 2020, when the agreement expired, there were 84,223 people waiting for inpatient or day case treatment, of whom 15,561 were waiting for more than 12 months, while 584,399 people were awaiting outpatient appointments, of whom 219,712 were waiting for more than 12 months.^4^ A further 35,878 people were waiting for inpatient or day case endoscopy appointments (NTPF, 2020).

This suggests that the addition of private hospital capacity could have been used to make progress on the public waiting lists, as the additional capacity was not fully needed to treat patients suffering from COVID-19. However, rather than being reduced, waiting lists actually increased, as the number waiting for inpatient/day case treatment in June 2020 was 17,518 (over 26%) higher than in February 2020 (when the first COVID-19 case was reported in Ireland), while the outpatient list had increased by 25,845 (over 4.6%) over the same period (NTPF, 2020). It should be noted that this was largely due to the cancellation of non-essential treatment, which was recommended by the National Public Health Emergency Team. However, it does suggest that, with greater foresight, provisions could have been made to accommodate other treatments in the additional capacity, rather than leaving it unutilised in the event of the non-materialisation of the anticipated surge.

It is acknowledged that this may have necessitated some amendments to the initial agreement, which was designed to provide surge capacity, although Clause 2.2.5 allowed for services to comprise ‘such further additional services as may be deemed clinically necessary, as agreed between Parties, having regard to the Common Purpose’ (Government of Ireland, 2020, p. 2), which could be open to interpretation. According to the Oireachtas (parliamentary) Special Committee on the COVID-19 Response, the HSE's stated objectives were ‘to provide a reserve for surge pressures, to maintain essential services for non-Covid healthcare and time dependent surgery and treatments…and to address the extensive build-up of delayed work as soon as possible’ (Special Committee on Covid-19 Response, 2020*b*, p. 71). However, the Committee noted that the National Public Health Emergency Team ‘did not lift the restriction on non-essential health care during the period of the contract’ (Special Committee on Covid-19 Response, 2020*b*, p. 72)., which would have meant that the Government would have had to over-rule this advice in order to avail of the additional capacity for non-COVID treatment. The Committee notes that in the case of any future surges, lessons should be learned ‘from the implementation of the first agreement and also regarding the use of additional capacity and facilities to reduce waiting lists’ (Special Committee on Covid-19 Response, 2020*b*, p. 72). This suggests a view by the Committee that the best use was not made of the additional capacity during the lifetime of the agreement.

From a longer-term perspective, the merger of the public and private hospital sectors would face obstacles, as over 46% of the population is covered by private health insurance (HIA, 2020*b*), a significant benefit of which is access to private hospitals. During the three months of the agreement between the HSE and private hospitals, the three open market insurers in Ireland made temporary arrangements to reflect the fact that private patients no longer had access to private hospitals (unless they were treated there as public patients) (HIA, 2020*a*). These involved rebates to members to reflect the fact that their insurance could no longer be used to garner treatment in private hospitals.

However, if a longer-term arrangement were to be put in place, this might lead to significant discontinuation of private health insurance, with a consequent additional demand on the public hospital system, which already has capacity constraints, as highlighted earlier. Indeed, it should be noted that the additional capacity envisaged under the Sláintecare plan assumed the presence of a private hospital system alongside the public system, albeit there was an expectation of reduced demand for private health insurance resulting from improved access to public hospitals (Committee on the Future of Healthcare, 2017).

Therefore, while some have wondered whether the pandemic could lead to a structural shift in the Irish healthcare system resulting in a single-tier hospital system, such as happened in the United Kingdom after the Second World War, the reality is that this remains unlikely. However, if the correct lessons are drawn from the first agreement with the private hospitals, then in the event of a further agreement being required, an opportunity might present itself to make inroads into Ireland's lengthy waiting times for public patients, which are the longest in Europe (Björnberg and Phang, 2019). There will need to be some significant and visible payoff to the State from any future agreement with the private hospitals, so this space will be watched with interest.

## Footnotes

1 Our account draws from the Heads of Terms of the agreement signed by the parties (Government of Ireland, 2020), our review of the national press (especially the *Irish Times*, *Sunday Business Post*, and *Irish Independent*), parliamentary debates and publications (Special Committee on Covid-19 Response, 2020*a*, 2020*b*) as well as informal conversations with officials in the Irish Medical Organisation (which represents doctors in Ireland) and the Irish Hospital Consultants Association (which represents hospital consultants in Ireland).

2 It should be noted that, in addition to these consultants who work fully in private practice, the vast majority of public hospital consultants also have private practice rights under their contracts, some of whom have rights to off-site (i.e., not on public hospital campuses) private practice (Independent Review Group, 2019). However, the agreement referred to here only covered fully private consultants.

3 The 2008 public hospital consultant contract agreement offers three types of contract. Type A involves treating public patients only, type B allows for an agreed maximum proportion of private practice, which must be carried out on public hospital campuses, and type C allows for off-site private practice.

4 The most recent figures show that, as of February 2021, the number waiting for inpatient/day case treatment had reduced to 80,936, but that the number waiting 12 months or more had increased to 22,832, while the outpatient waiting list had increased to 626,895, of whom 280,717 were waiting for 12 months of more (NTPF, 2021). However, recent analysis suggests that the way waiting times are collected in Ireland actually underestimates the length of time that patients are waiting for treatment and that Ireland's waiting times are actually longer than previously reported (Brick and Connolly, 2021).
